# From Thoracotomy to Robotic Platforms in Thoracic Surgery: Technical Evolution, Simulation-Based Training, and Surgical Outcomes

**DOI:** 10.3390/cancers18193193

**Published:** 2026-10-02

**Authors:** Michele Piazzolla, Doroty Sampietro, Marco Donatello Delcuratolo, Paolo Nicola Camillo Girotti, Lucia Anna Muscarella, Paola Parente

**Affiliations:** 1 Unit of Thoracic Surgery, Fondazione IRCCS Casa Sollievo Della Sofferenza, 71013 San Giovanni Rotondo, Italy; d.sampietro@operapadrepio.it (D.S.); p.girotti@operapadrepio.it (P.N.C.G.); 2 Medical Oncology Unit, Fondazione IRCCS Casa Sollievo Della Sofferenza, 71013 San Giovanni Rotondo, Italy; 3 Lung Cancer Research Unit, Fondazione IRCCS Casa Sollievo Della Sofferenza, 71013 San Giovanni Rotondo, Italy; l.muscarella@operapadrepio.it; 4 Unit of Pathology, Azienda Ospedaliera Universitaria Ospedali Riuniti, 71100 Foggia, Italy; paolaparente77@gmail.com

**Keywords:** thoracic surgery, video-assisted thoracic surgery, robotic-assisted thoracic surgery, simulation, surgical training, learning curve, non-small cell lung cancer

## Abstract

Thoracic surgery has progressively moved from open thoracotomy to video-assisted and robot-assisted approaches, reducing access-related trauma while changing the technical environment in which pulmonary resections are performed and taught. This narrative review examines the clinical evolution of open, video-assisted thoracic surgery (VATS), and robot-assisted thoracic surgery (RATS), with particular attention to the evidence supporting their perioperative and oncological outcomes, the learning curves associated with minimally invasive surgery, and the role of simulation-based training. Randomized trials now provide robust evidence that VATS improve postoperative recovery compared with thoracotomy in appropriately selected patients with predominantly early-stage non-small cell lung cancer, without compromising long-term oncological outcomes. Randomized comparisons between RATS and VATS support RATS as an oncologically acceptable minimally invasive alternative and suggest advantages in selected technical endpoints, particularly blood loss and lymph node retrieval, but do not demonstrate consistent superiority in major clinical or survival outcomes. Learning curves vary according to previous experience, case complexity, supervision, institutional volume, and the endpoint used to define competence, making fixed case-number thresholds an incomplete measure of proficiency. Simulation can improve selected technical skills and provide objective performance assessment, although evidence becomes progressively less robust when moving from simulator performance to operating room transfer and patient-level outcomes. Surgical approach, therefore, should be interpreted within a broader framework that includes patient and tumour characteristics, oncological quality, surgical expertise, institutional resources, and structured training.

## Simple Summary

Thoracic surgery has changed substantially with the introduction of video-assisted and robot-assisted techniques, which allow many lung operations to be performed through smaller incisions. These approaches can reduce the physical impact of surgery, but they also require surgeons to learn new technical skills and work through different operative interfaces. This review examines how open, video-assisted, and robotic thoracic surgery have developed, how their clinical results compare, and how surgeons are trained to use them safely. Particular attention is given to learning curves and simulation-based training. Current evidence supports minimally invasive surgery for appropriately selected patients with lung cancer, although technical advantages do not always translate into better clinical or long-term cancer outcomes. Simulation can improve selected surgical skills, but evidence that these improvements consistently translate into better operating room performance or patient outcomes remains limited.

## 1. Introduction

Thoracic surgery has developed within a distinctive set of anatomical and physiological constraints. The thorax is a closed, negative-pressure cavity containing the lungs, heart, and great vessels; safe surgery in this region requires not only technical precision, but also close integration with ventilation, circulation, anaesthesia, and perioperative physiology. Progress in thoracic surgery has therefore never depended on surgical instruments alone, but on the parallel development of imaging, anaesthesia, oncology, perioperative care, and operative judgment.

Over time, pulmonary surgery has moved from open thoracotomy toward video-assisted thoracic surgery (VATS) and robot-assisted thoracic surgery (RATS). This transition has reduced access-related trauma for many patients, but it has also changed the way surgeons visualize anatomy, manipulate tissue, and perform complex dissections. Minimally invasive thoracic surgery requires the surgeon to work through a screen, fixed ports, long or articulated instruments, and, in robotic surgery, a console-based interface. These changes have created new technical demands and have made learning curves, structured training, simulation, and objective assessment increasingly relevant.

At the same time, the adoption of less invasive or more technologically advanced approaches should not be equated automatically with improved clinical outcomes. Technical advantages, perioperative recovery, quality of lymph node assessment, economic impact, and long-term oncological outcomes represent different dimensions of surgical performance and are supported by different levels of evidence. This distinction is particularly important when comparing open surgery, VATS, and RATS, and when interpreting the role of simulation in surgical training.

This narrative review examines the evolution from open thoracotomy to VATS and RATS, with particular attention to the clinical evidence supporting these approaches and to the educational challenges created by minimally invasive techniques. It reviews learning curves and simulation-based training, distinguishing simulator validation and technical skill acquisition from transfer to operative performance and patient-level outcomes. Finally, it summarizes current evidence on perioperative, technical, economic, and long-term oncological outcomes in non-small cell lung cancer (NSCLC), while considering patient selection, tumour characteristics, surgical expertise, and institutional context.

## 2. Methods: Narrative Review Approach

This review was designed as a narrative synthesis of the literature rather than as a systematic review. The literature search was conducted primarily through PubMed/MEDLINE and was updated through September 2026. Additional studies were identified from the reference lists of relevant randomized trials, systematic reviews, meta-analyses, consensus documents, and major thoracic surgery guidelines.

The search was organized around three main areas reflecting the scope of the review: (1) the development and clinical validation of open, video-assisted, and robot-assisted thoracic surgery; (2) perioperative, technical, economic, and long-term oncological outcomes associated with the different surgical approaches; and (3) learning curves, simulation-based training, competency assessment, and transfer of technical skills to the clinical setting. Search terms were adapted to each topic and included combinations of terms related to thoracotomy, video-assisted thoracic surgery, uniportal VATS, robotic-assisted thoracic surgery, lung resection, complications, conversion, operative time, blood loss, lymph node dissection, nodal upstaging, costs, survival, learning curve, simulation, virtual reality, robotic training, competency assessment, and skill transfer.

Study selection was guided by clinical relevance and by the level of evidence available for each question. Randomized controlled trials (RCTs) and prospective comparative studies were given priority when evaluating differences between surgical approaches. Systematic reviews and meta-analyses were used to place individual trials in context and to assess the consistency of findings across studies. Large retrospective or registry-based studies were considered when randomized evidence was unavailable, particularly for uncommon clinical scenarios, advanced disease, or specific technical outcomes. Historical reports, narrative reviews, expert consensus documents, and guidelines were used mainly to describe the development of surgical techniques, define accepted surgical standards, and discuss training frameworks.

Evidence derived from different study designs was not considered equivalent. Randomized evidence was distinguished from observational comparisons, while pooled analyses were interpreted in relation to the quality, consistency, and heterogeneity of the studies they included. Technical advantages of a surgical platform were considered separately from demonstrated improvements in patient-centred or oncological outcomes.

For lymph node assessment, the absolute number of retrieved nodes was not considered in isolation. Nodal evaluation was interpreted according to the anatomical extent and completeness of dissection, the number and distribution of nodal stations examined, and the rate of pathological nodal upstaging. The terminology and principles of intraoperative nodal assessment were based on the European Society of Thoracic Surgeons (ESTS) definitions proposed by Lardinois et al. and interpreted in light of the more recent ESTS expert consensus on technical standards for segmentectomy by Brunelli et al., with particular attention to systematic or lobe-specific nodal dissection rather than lymph node count alone [1,2].

A similar distinction was applied to simulation-based training. Evidence of simulator validity, improvement in performance within the simulated environment, transfer of technical skills to non-simulated tasks, performance in the operating room, and patient-level outcomes were treated as separate levels of evidence. Particular attention was paid to whether reported improvements reflected better simulator performance or whether they had been shown to translate into measurable clinical performance.

Because this was a narrative review, no formal meta-analysis or standardized risk-of-bias assessment was performed. Nevertheless, study design, population, comparator, outcome definition, and major methodological limitations were considered when interpreting the available evidence and when drawing conclusions across studies.

## 3. Evolution and Clinical Validation of Thoracic Surgical Approaches

### 3.1. From Open Thoracotomy to Minimally Invasive Access

Open thoracotomy provided the operative exposure on which modern pulmonary resection was developed and remains indispensable when direct access, tactile feedback, complex reconstruction, or immediate vascular control are required. The evolution toward minimally invasive surgery did not make these principles obsolete; rather, it sought to reproduce the same anatomical and oncological operation while reducing the trauma required to access the chest. This transition has occurred alongside improvements in thoracic anaesthesia, imaging, stapling and energy devices, and perioperative care [3].

VATS changed the relationship between the surgeon and the operative field by replacing direct visualization and manual access with thoracoscopic imaging and long instruments introduced through fixed ports. Multiportal VATS became the dominant minimally invasive approach for anatomical lung resection, while uniportal VATS subsequently reduced access to a single intercostal incision. Robotic surgery introduced a further change in interface through three-dimensional visualization, articulated instruments, tremor filtration, and console control. These platforms differ substantially in ergonomics and technical capability, but platform characteristics alone cannot establish clinical superiority. The principal advantages, limitations, and current roles of the approaches discussed in this review are summarized in Table 1.

### 3.2. VATS: From Feasibility to Randomized Clinical Evidence

The clinical adoption of VATS lobectomy initially depended on feasibility studies and observational comparisons. Cancer and Leukemia Group B (CALGB) 39802 was a prospective multi-institutional feasibility study that enrolled 128 patients and demonstrated that standardized VATS lobectomy could be performed successfully across participating centres; among 111 patients with stage I disease, 96 underwent successful VATS lobectomy [4]. Although this study did not compare VATS with thoracotomy, it was important in establishing reproducibility beyond isolated expert series.

Randomized evidence subsequently addressed patient-centred outcomes. Bendixen et al. randomized 206 patients to VATS lobectomy or anterolateral thoracotomy and demonstrated less postoperative pain and better quality of life after VATS [5]. Long et al. later reported a multicentre randomized comparison in which 425 evaluable patients underwent VATS or axillary thoracotomy. VATS was associated with lower blood loss and shorter operative time, while lymph node yield, length of stay, morbidity, mortality, and margin status were similar between groups [6].

The VIOLET trial provided the largest randomized comparison of VATS and open lobectomy in early-stage lung cancer. Among 503 randomized patients, VATS produced better recovery of physical function at 5 weeks, shorter hospitalization, and fewer serious adverse events, while measures of surgical and early oncological quality were comparable [7]. Long-term survival was not the primary endpoint of the individual trial, and its early survival estimates should therefore not be interpreted as a definitive oncological comparison.

That limitation was addressed by the 2026 individual-patient-data meta-analysis by Harris et al., which combined the three eligible randomized trials and included 1185 patients with clinical early-stage NSCLC. Overall survival favoured VATS (hazard ratio [HR] 0.79, 95% confidence interval [CI] 0.65–0.96), whereas disease-free survival was similar (HR 0.91, 95% CI 0.75–1.12) [8]. These findings provide the strongest available evidence that VATS do not compromise long-term cancer outcomes in patients who are suitable for either approach. Their generalizability, however, remains greatest for trial-eligible, predominantly early-stage populations.

Evidence for uniportal VATS has developed mainly through propensity-adjusted cohorts and meta-analyses rather than large randomized trials. Available studies generally report perioperative and oncological outcomes comparable with multiportal VATS in selected patients, but the evidence hierarchy is lower and should not be interpreted as proof of superiority of a single-incision approach [9,10].

### 3.3. RATS: Technical Capability and Demonstrated Clinical Benefit

RATS offer several technical characteristics that are attractive in thoracic surgery, including three-dimensional visualization, articulated instruments, tremor filtration, and independent camera control. These features can facilitate dissection in confined spaces and may be particularly useful around hilar structures and mediastinal lymph nodes. However, technical capability must be distinguished from demonstrated improvement in clinical outcomes.

The ROMAN multicentre randomized study compared robotic and manual VATS major lung resection. Seventy-seven patients were randomized, and 71 were included in the final analysis. The study did not demonstrate a broad perioperative superiority of RATS, although the robotic approach was associated with a more extensive lymph node assessment in selected metrics [11]. The RVlob randomized trial subsequently enrolled 320 patients and showed similar short-term safety and postoperative recovery between robotic and VATS lobectomy, with differences in selected technical outcomes and higher hospitalization costs for the robotic approach [12].

RAVAL extended the comparison to health utility and economic outcomes. Of 186 randomized patients, 164 were analysed. Health utility was broadly comparable between approaches; RATS was associated with a modest increase in sampled lymph nodes and an incremental cost-effectiveness ratio of approximately USD 14,926 per quality-adjusted life-year in the trial model [13]. Such economic findings are context-dependent and should not be generalized across healthcare systems with different acquisition, utilization, and reimbursement models.

Long-term randomized evidence is now available from RVlob. After a median follow-up of approximately 58 months, three-year overall survival was 94.6% after RATS and 91.5% after VATS, with comparable disease-free survival; the prespecified conclusion was non-inferiority rather than superiority [14]. A 2026 systematic review and meta-analysis restricted to five randomized trials and 712 patients similarly found no significant difference in overall complications, conversion, operative time, or three-year overall survival. RATS was associated with lower blood loss and a higher lymph node yield, but also with higher hospitalization costs [15].

### 3.4. Interpreting the Evolution of Surgical Access

The transition from thoracotomy to VATS and RATS should therefore not be interpreted as a linear hierarchy in which each newer platform is inherently superior to the previous one. The most robust randomized evidence establishes a recovery advantage for VATS over thoracotomy in appropriately selected early-stage patients without loss of oncological effectiveness. For RATS versus VATS, randomized evidence supports comparable safety and oncological outcomes, with selected technical differences whose clinical magnitude and economic relevance remain context-dependent.

The distinction between technical capability and demonstrated patient benefit is particularly important as increasingly complex procedures are approached minimally invasively. Tumour location, extent of resection, prior treatment, vascular or bronchial involvement, patient physiology, surgeon experience, and institutional resources may all influence the safest access. The pivotal and high-level studies underpinning the current clinical validation of minimally invasive lung resection are summarized in Table 2.

## 4. Evolution of Thoracic Surgical Training and Learning Curves

### 4.1. The Traditional Apprenticeship Model and Its Limitations

Thoracic surgical training has traditionally followed an apprenticeship model in which trainees progress from observation to assistance and then to the supervised performance of increasingly complex parts of an operation. This model remains essential because operative judgment, anatomical interpretation, management of unexpected findings, and decision-making during complications cannot be reproduced completely outside the operating room.

The transition to minimally invasive surgery has nevertheless changed the technical environment in which these skills are acquired. In VATS, the surgeon works through fixed ports using long instruments and indirect visualization, with reduced tactile feedback and a greater dependence on camera orientation and assistant coordination [16,17]. Robotic surgery modifies the operative interface further by introducing console control, articulated instruments, three-dimensional visualization, clutching, camera control, and a physical separation between the console surgeon and the bedside team [18].

These changes have increased the value of structured technical training and objective assessment. Operative volume remains an important component of experience, but case numbers alone do not describe the complexity of the procedures performed, the degree of trainee autonomy, the quality of supervision, or the technical endpoints achieved [19,20].

### 4.2. Learning Curves in VATS and Uniportal VATS

The learning curve for VATS lobectomy has been investigated using several endpoints, including operative time, conversion, complications, blood loss, technical performance, and oncological quality. Early studies suggested that relevant improvements often occur during the first several dozen procedures [21,22]. This has led to the frequently quoted estimate of approximately 50 cases for the initial VATS lobectomy learning curve.

Such a threshold should be interpreted cautiously. In a large analysis of 24,196 VATS lobectomies from the Society of Thoracic Surgeons General Thoracic Surgery Database, Puri et al. found that most high-volume centres fulfilled predefined proficiency criteria within approximately 50 cases, but different learning patterns were observed and proficiency was not necessarily maintained once achieved [23]. The number of procedures required to reach a plateau therefore depends on the endpoint used to define proficiency and on the setting in which the procedure is learned.

This issue becomes particularly relevant when surgeons move from multiportal to uniportal VATS. Uniportal access places the camera, instruments, staplers, and suction through the same incision, making instrument alignment, exposure, and coordination between surgeon and assistant especially important. Previous experience with multiportal VATS can substantially influence this transition.

Laven et al. analysed the learning curve for uniportal VATS lobectomy in surgeons with previous thoracoscopic experience and reported a reduction in technique-related complications after approximately 50–96 procedures [24]. Chen et al., studying uniportal anatomical segmentectomy, identified an earlier phase of technical proficiency in surgeons who already had extensive experience with multiportal VATS, followed by a later phase of greater procedural maturity [25]. These findings support the concept that learning curves are strongly influenced by previous experience and should not be interpreted as fixed procedural thresholds.

For this reason, competence and case volume should not be treated as interchangeable. Objective technical assessment tools may complement operative numbers by evaluating specific aspects of performance and identifying whether improvement is maintained over time [19,20].

### 4.3. Learning Curves in Robotic Thoracic Surgery

Robotic thoracic surgery introduces a different set of technical demands. Familiarity with minimally invasive anatomical dissection remains relevant, but console control, wristed instrumentation, camera management, energy use, clutching, and coordination with the bedside assistant require platform-specific training [18].

As with VATS, the number of cases required to overcome the robotic learning curve varies according to the endpoint examined. Published series and pooled analyses generally identify the greatest early improvement within the first several dozen procedures, but operative time, conversion, lymph node assessment, and complication rates may reach stability at different points.

More recent evidence also suggests that robotic learning may be multiphasic rather than characterized by a single threshold. Qu and Harling reported an early competency phase at approximately 20 robotic anatomical lung resections and a later proficiency phase around 60 cases, with further improvement beyond the initial plateau [26]. These estimates are useful for understanding the progression of technical performance but should not be regarded as universal credentialing thresholds. Surgeon background, institutional volume, case complexity, proctoring, bedside-team experience, and the definition of the endpoint all influence the observed curve.

This distinction is particularly important in thoracic surgery, where efficiency and technical fluency do not necessarily imply oncological completeness. A surgeon may reduce operative time before reaching consistent proficiency in lymph node dissection or management of technically demanding hilar anatomy. Learning curves should therefore be interpreted using multiple endpoints rather than a single measure of procedural speed.

### 4.4. From Case Numbers to Competency-Based Progression

The available evidence suggests that learning curves are better considered as tools for monitoring performance than as fixed numerical requirements for independent practice. Case numbers remain useful because repeated clinical exposure is necessary, but they provide only an indirect measure of competence.

Competency-based progression combines operative exposure with objective assessment, structured feedback, and predefined technical standards [17,20]. Procedure-specific assessment tools, proficiency-based training, and methods such as cumulative-sum analysis may therefore provide a more informative picture of progression than operative volume alone.

Simulation fits naturally within this framework. Its potential value lies not in replacing supervised surgery, but in allowing selected technical components to be practised and assessed before or alongside clinical exposure. The different simulation modalities used in thoracic surgery, and the distinction between simulator performance and transfer to clinical practice, are discussed in the following section.

## 5. Simulation-Based Training in Thoracic Surgery

### 5.1. Simulation Models in Thoracic Surgery

Simulation has progressively been incorporated into thoracic surgical training to provide repeated practice of technical tasks outside the operating room. Its role is particularly relevant for minimally invasive procedures, in which restricted access, indirect visualization, limited tactile feedback, and the potential consequences of vascular injury make some technical steps difficult to learn through clinical exposure alone. An international Delphi consensus identified VATS lobectomy, VATS segmentectomy, mediastinal lymph node dissection, flexible bronchoscopy, and robotic port placement and docking among the procedures considered most suitable for simulation-based training in thoracic surgery [27].

Available models range from relatively simple box trainers to anatomically structured physical models, wet-lab preparations, and virtual-reality platforms. These modalities should not be considered interchangeable because they reproduce different components of the operation.

Early physical simulators were designed primarily to reproduce the geometry of thoracoscopic surgery. Iwasaki et al. developed an anatomical trainer incorporating artificial lung, bronchial, and vascular structures through which trainees could practise the principal steps of VATS lobectomy [28]. More sophisticated synthetic models subsequently attempted to reproduce tissue characteristics as well as anatomy. The polyvinyl-alcohol hydrogel model described by Sato and Morikawa, for example, provides replaceable lung structures within an anatomical thoracic cage and permits dissection, stapling, and vessel manipulation using standard thoracoscopic instruments [29].

The Stupnik VATS simulator follows a similar task-oriented approach. Its anatomical lung models allow complex procedures to be divided into individual exercises involving lung manipulation, energy devices, stapling, vascular dissection, and anatomical resection [30]. Breaking an operation into repeatable components may allow trainees to practise difficult manoeuvres before integrating them into a complete procedure.

Wet-lab and animal models offer more realistic tissue handling and can reproduce a complete operative sequence. Their use, however, is limited by cost, availability, logistical requirements, infection-control issues, and ethical considerations [31]. These limitations make them better suited to selected stages of training than to frequent repetitive practice.

Virtual reality (VR) simulation offers different advantages. Tasks can be repeated without consumable materials, and computer-generated metrics can be used to evaluate completion time, errors, instrument movement, and other aspects of technical performance. Dedicated VATS modules have also been developed for procedural assessment. Jensen et al., for example, demonstrated validity evidence for a virtual-reality VATS lobectomy test capable of distinguishing different levels of previous operative experience [32].

The availability of multiple simulation modalities therefore offers the possibility of matching the training platform to the skill being taught rather than seeking a single simulator capable of reproducing the entire operation.

### 5.2. Robotic Simulation

Robotic surgery has created an additional need for simulation because the surgeon must become familiar with a new operative interface before assuming clinical responsibility at the console. Basic skills include camera manipulation, clutching, instrument control, energy activation, coordination of articulated instruments, and communication with the bedside assistant [18,33].

Virtual robotic platforms, including the Da Vinci Skills Simulator and more recent integrated simulation systems, allow these skills to be practised without occupying operating room time or exposing patients to the earliest part of the learning process. Performance can be recorded using automated metrics, providing trainees and instructors with objective information on progression [34,35].

Evidence from randomized studies across robotic surgical specialties indicates that VR training improves simulator-based objective scores and task completion when compared with no additional training. A systematic review and meta-analysis by Kawashima et al. found improvements in both objective performance scores and task time, while VR and dry-lab training produced broadly comparable results for several basic robotic skills [36]. These data support VR as a tool for early robotic skill acquisition, although much of the underlying evidence is not specific to thoracic surgery.

Physical robotic simulators address aspects that virtual environments reproduce less effectively, particularly tissue manipulation and force control. Gamberini et al. developed a high-fidelity sensorized vascular model capable of measuring the strain applied during robotic vessel manipulation and providing real-time feedback [37]. Face, content, and construct validity were explored in a preliminary cohort, but the study did not assess subsequent clinical performance. This distinction is important when interpreting simulator studies.

Wet-lab training also remains part of several robotic curricula because it allows console skills to be integrated with tissue dissection and team workflow. In practice, robotic training is therefore increasingly multimodal, combining virtual exercises, physical models, supervised laboratory experience, bedside participation, and progressively more demanding console exposure [38].

### 5.3. Simulator Validation, Skill Acquisition, and Clinical Transfer

The increasing availability of simulators raises a separate question: whether a model that appears realistic or improves simulator scores also improves clinical performance.

These are different levels of evidence. A simulator may demonstrate validity because its content is considered appropriate, because experienced surgeons perform differently from novices, or because its measurements are reproducible. Such findings support the simulator as an assessment or training tool but do not by themselves demonstrate transfer to the operating room.

This distinction remains a limitation of the thoracic simulation literature. Whittaker et al. identified 33 thoracic surgical simulators reported in 31 publications. Evidence supporting at least one component of validity was available for fewer than half of the models, and comprehensive validation was uncommon [39]. The number of available simulators therefore exceeds the number for which robust educational validation has been demonstrated.

Thoracic-specific training studies nevertheless provide evidence that structured practice can improve measurable technical skills. Koliakos et al. prospectively evaluated seven first-year thoracic surgery residents undergoing a six-month VATS simulation programme using two dry-lab platforms. Instrument travel distance, task completion time, and periods of extreme motion improved after training, while the untrained control group showed no comparable change [40]. These results demonstrate improvement in technical dexterity but should not be interpreted as direct evidence of better intraoperative or patient outcomes.

The next step in the evidence pathway is skill transfer. Earlier systematic evidence from surgical education showed that proficiency-based simulation training can improve subsequent performance in patient-based settings when incorporated into structured programmes [41]. For robotic surgery, Schmidt et al. also reported evidence that technical skills acquired using VR simulation can transfer to operative performance, although the available studies were limited in number and heterogeneous [42].

More recent evidence supports a cautious interpretation. Sarmento et al. found that VR robotic simulation improves proximal non-virtual technical performance, while evidence for equivalence with non-virtual training and for downstream patient-level benefit remains less certain [43]. Similarly, a systematic review focused specifically on robotic-assisted thoracic surgery found that simulation supports familiarization, skills development, and team education, but that translation of these improvements into clinical practice remains incompletely demonstrated [44].

Recent randomized evidence also illustrates the uncertainty surrounding transfer. In a 2026 meta-analysis restricted to randomized studies evaluating transfer from robotic VR simulation to non-virtual performance, the pooled effect on global technical skills favoured simulation but did not reach statistical significance, with wide confidence intervals and small study populations [45].

The available evidence can therefore be interpreted as a progression rather than a single endpoint: simulator validity → improvement during simulation → transfer to non-virtual technical tasks → improvement in operative performance → patient-level benefit. Evidence is strongest for the first two steps and becomes progressively less robust toward clinical and patient-level outcomes.

### 5.4. Simulation Within Structured Training Curricula

The educational value of simulation depends not only on the fidelity of the model but also on how it is incorporated into training. Repeated practice without defined objectives does not necessarily establish competence. Simulation is more informative when combined with predefined proficiency targets, objective assessment, faculty feedback, and subsequent supervised operative exposure [17,41].

This concept is increasingly reflected in contemporary thoracic surgical recommendations. The 2025 Society of Thoracic Surgeons expert consensus on robotic training proposed a standardized framework covering programme expectations, training components, assessment, and feedback [38]. Simulation forms part of this pathway but is not presented as a substitute for clinical experience or as an independent method of certification.

The same caution applies to automated performance metrics. Simulator-generated scores can document progression and help identify specific technical difficulties, but they measure only selected components of surgical competence. Anatomical interpretation, decision-making, management of unexpected events, communication, and operative judgment still require supervised clinical assessment.

Simulation may also have a role beyond individual technical performance. Robotic surgery separates the console surgeon from the bedside team and requires coordinated responses to docking problems, vascular emergencies, conversion, and other uncommon events. Multidisciplinary simulation may therefore be useful for team communication and organisational preparedness, although evidence linking such training to patient outcomes remains limited [44].

Artificial intelligence and automated performance analysis may further expand the ability of simulation platforms to identify recurrent errors and individualize training. At present, however, these applications remain largely developmental. Their educational value should be established through external validation and demonstration of meaningful transfer rather than inferred from technical capability alone.

Overall, simulation should be regarded as an adjunct to supervised operative training rather than as an alternative to it. Current evidence supports its role in deliberate practice, objective assessment, and early skill acquisition, while evidence for improved independent operative performance and patient-level outcomes remains less mature. The principal simulation modalities and the level of evidence supporting their use are summarized in Table 3.

## 6. Short-Term Surgical Outcomes of Minimally Invasive Thoracic Surgery in NSCLC

### 6.1. Postoperative Recovery and Complications

Although long-term survival remains the principal oncological endpoint after lung cancer surgery, short-term outcomes are clinically relevant because they influence postoperative pain, mobilization, respiratory recovery, length of stay, and the ability to proceed with subsequent treatment when indicated.

The strongest randomized evidence concerns the comparison between VATS and thoracotomy. Bendixen et al. showed that VATS lobectomy was associated with less postoperative pain and better quality of life than anterolateral thoracotomy in patients with early-stage lung cancer [5]. The VIOLET trial subsequently confirmed a benefit in early recovery. Among 503 randomized patients, physical function at 5 weeks was better after VATS, median hospital stay was shorter, and serious postoperative adverse events were less frequent than after open surgery [7].

These findings are consistent with broader comparative evidence suggesting lower postoperative morbidity after minimally invasive resection than after thoracotomy [46]. Nevertheless, complication rates should not be attributed to surgical access alone. Patient selection, extent of resection, enhanced recovery pathways, institutional experience, and thresholds for conversion can substantially influence perioperative outcomes.

The comparison between RATS and VATS is less marked. The ROMAN randomized study found no broad perioperative superiority of RATS, while RVlob reported comparable postoperative recovery and complication rates between robotic and thoracoscopic lobectomy [11,12]. In the 2026 meta-analysis restricted to randomized trials, overall postoperative complications and conversion to thoracotomy were not significantly different between RATS and VATS [15]. Current randomized evidence therefore supports both approaches as established minimally invasive options rather than demonstrating a consistent perioperative superiority of one over the other.

### 6.2. Operative Time, Blood Loss, and Conversion

Operative time is frequently used as a measure of surgical efficiency, but its interpretation is particularly sensitive to surgeon experience, case complexity, operative definitions, and institutional workflow. Long et al. reported a shorter operative time with VATS than with axillary thoracotomy [6], whereas the VIOLET health-technology assessment found greater theatre time for VATS despite shorter postoperative hospitalization and favourable overall resource use [47]. These differences illustrate why operative time should not be used in isolation as a measure of clinical effectiveness.

A similar pattern is observed when RATS is compared with VATS. Individual studies have reported differences in either direction, but the 2026 meta-analysis of randomized trials found no significant difference in pooled operative time between the two techniques [15].

Blood loss appears to be one of the technical outcomes for which a difference between RATS and VATS is more consistently observed. In RVlob, intraoperative blood loss was lower in the robotic group [12]. In the randomized-trial meta-analysis by Ren et al., RATS was associated with a mean reduction in blood loss of approximately 62 mL compared with VATS [15]. Whether a difference of this magnitude has clinical relevance in an uncomplicated anatomical lung resection remains uncertain, but it may reflect differences in visualization and instrument control.

Conversion to thoracotomy should also be interpreted cautiously. Conversion is not necessarily a failure of minimally invasive surgery; in some circumstances it represents an appropriate safety decision in response to bleeding, difficult hilar anatomy, adhesions, or oncological findings. In randomized comparisons of RATS and VATS, conversion rates have been broadly similar, and pooled randomized evidence has not demonstrated a significant difference [11,15].

This may be different in technically more demanding populations. In a recent multicentre retrospective study of patients undergoing resection after neoadjuvant chemoimmunotherapy, conversion was less frequent after RATS than after VATS [48]. The finding is clinically interesting, particularly because post-induction surgery may involve more fibrosis and challenging hilar dissection, but it should not be generalized to routine lung resection. The study was non-randomized, the two treatment groups were markedly unbalanced in size, and operative approach was influenced by institutional practice.

### 6.3. Lymph Node Assessment, Upstaging, and Downstaging

Lymph node assessment requires separate consideration because it is both a component of pathological staging and an indicator of the oncological quality of lung resection. The absolute number of retrieved lymph nodes is informative but does not provide a complete measure of the quality of lymphadenectomy.

The ESTS guidelines for intraoperative lymph node staging distinguish nodal sampling from systematic and lobe-specific nodal dissection and recommend systematic nodal assessment as part of complete oncological resection [1]. These principles remain relevant in contemporary minimally invasive surgery. The more recent ESTS consensus on technical standards for segmentectomy similarly recommends systematic or lobe-specific lymph node dissection rather than reliance on node count alone [2].

Earlier comparative evidence raised concerns that thoracoscopic lobectomy could retrieve fewer lymph nodes, particularly mediastinal nodes, than open surgery [49]. Randomized evidence has subsequently provided a more reassuring picture. In VIOLET, pathological nodal upstaging was similar after VATS and open surgery [7]. This suggests that, when performed according to oncological principles, thoracoscopic access does not necessarily compromise pathological staging.

RATS has shown differences in selected nodal metrics when compared with VATS. In the ROMAN trial, robotic surgery was associated with a greater number of sampled nodal stations and more hilar and mediastinal nodes [11]. A modest increase in lymph node yield was also reported in RVlob and RAVAL [12,13], and the meta-analysis restricted to RCTs by Ren et al. found a pooled increase of approximately 2.6 retrieved nodes with RATS [15].

However, greater lymph node yield should not automatically be interpreted as improved oncological effectiveness. Nodal upstaging is a related but distinct endpoint and is influenced by the quality of preoperative staging, prevalence of occult nodal disease, pathological processing, patient selection, and the surgical dissection itself.

A 2026 network meta-analysis including 30 studies and more than 71,000 patients with clinical N0 lung cancer found differences among open, VATS, and robotic approaches in both N1 and N2 upstaging [50]. Open surgery was associated with more cN0-to-pN1 upstaging than either minimally invasive approach, while both open surgery and RATS showed more cN0-to-pN2 upstaging than VATS. These findings suggest possible differences in nodal evaluation, but they arise from a heterogeneous evidence base that includes indirect and predominantly non-randomized comparisons. They should therefore not be interpreted as proof that one access route provides superior oncological staging in all patients.

Downstaging requires a different interpretation. In patients receiving neoadjuvant treatment, pathological nodal downstaging, major pathological response, and pathological complete response primarily reflect tumour biology and response to systemic therapy rather than the surgical access used for resection. In the multicentre series reported by Srivatsa et al. after neoadjuvant chemoimmunotherapy, lymph node downstaging, major pathological response, pathological complete response, R0 resection, and lymph node harvest were comparable between RATS and VATS [48]. These endpoints are therefore useful for assessing whether minimally invasive surgery preserves resection quality after induction treatment, but they should not be used as direct measures of the intrinsic effectiveness of the surgical platform.

### 6.4. Economic Considerations

Cost comparisons require the same caution as technical outcomes because different studies measure different components of expenditure. Operative costs, disposable instruments, platform acquisition and depreciation, hospitalization, readmission, and total healthcare costs are not equivalent outcomes.

The economic analysis performed within VIOLET is informative in this regard. VATS required more theatre resources than open surgery, but this was offset by shorter hospitalization and lower subsequent healthcare utilization. Over 12 months, mean total costs were numerically lower after VATS while quality-adjusted life-years were greater [47]. In the UK National Health Service model, VATS was therefore highly likely to be cost-effective, but this result should not be assumed to apply unchanged to other healthcare systems.

The economic comparison between RATS and VATS is more complex. In RVlob, hospitalization costs were substantially higher in the robotic group [12]. The randomized-trial meta-analysis by Ren et al. also found higher hospitalization costs with RATS, largely related to equipment-associated expenditure [15].

RAVAL provides a different but complementary perspective. Although robotic surgery involved additional costs, the trial incorporated health utility into the analysis and estimated an incremental cost-effectiveness ratio of approximately USD 14,926 per quality-adjusted life-year [13]. This does not contradict studies reporting higher direct costs for robotic surgery. Rather, it illustrates the distinction between a procedure being more expensive and its being cost-effective within a particular healthcare and economic model.

Platform utilization, institutional case volume, acquisition or leasing arrangements, operating room efficiency, disposable costs, length of stay, and reimbursement can all change the economic balance. Cost should therefore be reported as a context-dependent outcome rather than as an intrinsic characteristic of the surgical technique.

Taken together, the current evidence indicates that the clearest perioperative advantage of minimally invasive surgery remains the reduction in access-related morbidity compared with thoracotomy. Differences between RATS and VATS are smaller and relate mainly to selected technical parameters, nodal assessment, and economic considerations. These comparisons should remain distinct from long-term oncological effectiveness, which is discussed in the following section. The main short-term findings are summarized in Table 4.

## 7. Long-Term Oncological Outcomes of Minimally Invasive Thoracic Surgery in NSCLC

Long-term oncological outcomes are central to the evaluation of minimally invasive lung cancer surgery. Overall survival (OS), disease-free survival (DFS), recurrence-free survival (RFS), recurrence patterns, and completeness of resection have all been used to compare surgical approaches. Interpretation of these data, however, requires particular attention to study design. Much of the earlier literature consists of retrospective series or propensity-adjusted comparisons, in which selection of the surgical approach may be associated with tumour stage, anatomical complexity, patient characteristics, institutional expertise, and the period in which surgery was performed.

For this reason, randomized evidence should be considered separately from observational comparisons.

### 7.1. VATS and Open Surgery

Earlier observational studies and meta-analyses generally reported either similar or favourable long-term survival after VATS compared with thoracotomy, but these analyses were predominantly based on non-randomized evidence [51]. The VIOLET trial provided randomized evidence that the early recovery benefits of VATS were not accompanied by an evident early oncological disadvantage. At the initially reported follow-up, progression-free and overall survival did not differ significantly between VATS and open lobectomy, although the individual trial was not powered to provide a definitive survival comparison [7].

The most informative long-term evidence is now provided by the individual-patient-data meta-analysis published by Harris et al. in 2026. Individual data from the three eligible randomized trials comparing VATS with open lobectomy were obtained for 1185 patients with clinical early-stage NSCLC. Overall survival favoured VATS, with a pooled hazard ratio of 0.79, while disease-free survival was similar between approaches (HR 0.91) [8].

This result represents a substantial advance over previous observational comparisons because it is derived entirely from randomized populations. Nevertheless, its interpretation should remain linked to the population studied. The trials predominantly included patients with early-stage disease who were considered suitable for lobectomy through either access. The findings therefore provide strong evidence that VATS does not compromise long-term oncological outcomes in appropriately selected patients, but they should not be extrapolated automatically to locally advanced tumours, complex bronchovascular resections, or post-induction procedures.

Comparisons within minimally invasive surgery address somewhat different questions. Studies comparing uniportal and multiportal VATS have generally not demonstrated a consistent difference in long-term oncological outcomes [9,10]. Similarly, comparisons between VATS segmentectomy and VATS lobectomy should be interpreted primarily as evidence regarding the extent of pulmonary resection rather than the surgical access itself [52]. These data therefore provide useful context but should remain distinct from comparisons of VATS with thoracotomy.

### 7.2. RATS and Other Surgical Approaches

The long-term evidence for robotic surgery has historically been more dependent on observational data. Randomized evidence is now available. In the long-term analysis of the RVlob trial, 320 patients with resectable stage I-IIIA NSCLC had been randomized to RATS or VATS lobectomy. After a median follow-up of approximately 58 months, three-year OS was 94.6% after RATS and 91.5% after VATS. RATS met the predefined criterion for non-inferiority. Three-year DFS was also comparable [14]. These data provide direct evidence that robotic lobectomy can achieve oncological outcomes comparable with VATS, but they do not demonstrate a survival advantage of the robotic approach.

This interpretation is consistent with more recent pooled randomized evidence. In the meta-analysis of randomized RATS-VATS trials by Ren et al., no significant difference in three-year overall survival was demonstrated [15]. Thus, current randomized evidence supports oncological equivalence or non-inferiority rather than superiority of RATS over VATS.

Observational comparisons remain useful for populations that are under-represented in randomized trials, but their findings require more cautious interpretation. Yang et al. compared robotic, VATS, and open lobectomy in matched patients with clinical stage I NSCLC. Five-year OS was similar among the three groups. Although an unadjusted difference in DFS was observed between RATS and VATS, surgical approach was not independently associated with either OS or DFS in multivariable analysis [53].

Evidence in more advanced disease is less extensive. Zirafa et al. retrospectively compared robotic and open anatomical lung resection in 131 patients with locally advanced NSCLC. Five-year OS was approximately 34% after robotic surgery and 31% after thoracotomy, with no significant differences in local recurrence-free or metastasis-free survival [54]. These findings support the feasibility of a robotic approach in selected patients with locally advanced disease, but the retrospective design, single-centre setting, and potential selection of technically suitable cases prevent conclusions regarding comparative superiority.

Large database analyses and systematic reviews have also reported broadly comparable survival after open, VATS, and robotic resection. Some have suggested better DFS or OS with a robotic approach in selected comparisons [51,55]. Such findings should be interpreted as hypothesis-generating. Differences in surgical era, institutional volume, staging practices, perioperative care, adjuvant treatment, and selection for a minimally invasive approach remain difficult to eliminate completely in non-randomized comparisons.

### 7.3. Interpreting Oncological Equivalence and Apparent Survival Differences

The available literature therefore supports a more cautious interpretation than a simple ranking of surgical approaches. For VATS versus thoracotomy, the evidence has progressed from retrospective comparisons to randomized trials and, most recently, an individual-patient-data meta-analysis of randomized studies. In patients with early-stage NSCLC suitable for either approach, the available evidence no longer supports a concern that VATS compromises long-term cancer control [8].

For RATS versus VATS, the evidence base is more recent. Randomized data demonstrate non-inferior or comparable survival, while some observational studies report differences favouring robotic surgery in selected endpoints. At present, these observational signals should not be interpreted as evidence of a general oncological superiority of the robotic platform [14,15].

It is also important to distinguish surgical access from the quality of the cancer operation. Complete resection, adequate lymph node assessment, appropriate resection margins, pathological stage, tumour biology, receipt of perioperative systemic therapy, and management of recurrence are likely to have a greater direct influence on long-term outcome than the choice of minimally invasive platform itself.

Finally, the populations represented in comparative studies are not uniform. Evidence is strongest for lobectomy in early-stage NSCLC, whereas patients with locally advanced disease, central tumours, post-induction fibrosis, sleeve resections, or other technically complex procedures remain less well represented in randomized comparisons. Conclusions regarding long-term equivalence should therefore remain linked to patient selection, tumour characteristics, procedural complexity, and surgical expertise. The principal studies informing the long-term oncological comparison of open, VATS, and robotic lung resection are summarized in Table 5.

## 8. Limitations of the Available Evidence

The evidence reviewed in this article has several limitations that should be considered when comparing open, video-assisted, and robotic thoracic surgery. First, the strength of evidence is not uniform across surgical approaches or outcomes. Randomized trials are available for selected comparisons, particularly VATS versus thoracotomy and, more recently, RATS versus VATS, but much of the literature still consists of retrospective institutional series, registry analyses, propensity-adjusted studies, and meta-analyses that combine heterogeneous study designs. Differences in patient selection, tumour stage, operative complexity, surgeon experience, perioperative pathways, and definitions of individual outcomes can therefore influence apparent differences between techniques.

Generalizability is another important limitation. Most randomized comparisons have focused on patients with predominantly early-stage NSCLC who were considered technically suitable for more than one surgical approach. Evidence is less robust for central tumours, locally advanced disease, post-induction resections, sleeve procedures, vascular reconstruction, and other technically demanding scenarios. In addition, many studies originate from experienced or high-volume minimally invasive centres. Outcomes obtained in these settings may not be reproduced during early institutional adoption or in centres with lower procedural volume.

The interpretation of technical endpoints also requires caution. Operative time, blood loss, conversion, lymph node yield, and nodal upstaging are influenced by factors other than the surgical platform itself. In particular, lymph node count should not be considered synonymous with the completeness of oncological nodal assessment, and pathological downstaging after neoadjuvant therapy primarily reflects treatment response and tumour biology rather than surgical access. Similarly, higher procedural costs do not necessarily imply lower cost-effectiveness, as economic results depend strongly on platform utilization, healthcare organization, length of stay, reimbursement, and the time horizon of the analysis.

Limitations are also evident in the literature on surgical training and simulation. Learning-curve thresholds vary according to previous experience, case complexity, supervision, institutional volume, and the endpoint used to define competence. Simulation studies provide relatively consistent evidence of improvement in selected technical skills, but simulator validation remains incomplete for many platforms, and evidence becomes progressively less robust when moving from simulator performance to transfer into the operating room and, ultimately, to patient-level outcomes. These limitations support a cautious interpretation of simulation as part of a structured training pathway rather than as a stand-alone measure of clinical competence.

Finally, the present article is a narrative review. Although the literature search was structured and greater weight was given to randomized trials, systematic reviews, meta-analyses, and contemporary consensus documents, no formal risk-of-bias assessment or quantitative synthesis was performed. The review should therefore be interpreted as a critical synthesis of the available evidence rather than as a systematic or meta-analytic estimate of comparative treatment effects.

## 9. Conclusions

The evolution of thoracic surgery should not be viewed simply as a progression from larger incisions to increasingly sophisticated minimally invasive platforms. Open thoracotomy, VATS, and robotic surgery provide different operative interfaces, and their value depends on the clinical setting in which they are used. Thoracotomy remains necessary in selected complex resections, while minimally invasive approaches have reduced the morbidity associated with surgical access and have become central to the treatment of resectable lung cancer.

The strength of the supporting evidence, however, differs between approaches. For patients with predominantly early-stage NSCLC who are suitable for either access, randomized evidence supports VATS over thoracotomy in terms of postoperative recovery without evidence of compromised long-term oncological outcomes. Robotic surgery represents an established minimally invasive alternative to VATS and may offer advantages in selected technical endpoints, including visualization, instrument articulation, blood loss, and lymph node retrieval. Current randomized evidence, however, supports oncological comparability or non-inferiority rather than a general clinical or survival superiority of RATS over VATS. Surgical access should therefore not be considered an oncological endpoint in itself. Complete resection, appropriate margins, systematic nodal assessment, patient and tumour selection, and sound intraoperative judgment remain fundamental regardless of the platform used.

Technical evolution has also changed how thoracic surgeons should be trained. Learning curves cannot be reduced reliably to a fixed number of procedures, because previous experience, supervision, case complexity, institutional volume, and the endpoint used to define competence all influence progression. Simulation provides a controlled environment for deliberate practice, objective assessment, and early technical skill acquisition, and its role is increasingly incorporated into structured thoracic and robotic training pathways. Nevertheless, evidence is strongest for improvement in simulator-based or proximal technical performance and becomes less robust when clinical transfer and patient-level outcomes are considered. Simulation should therefore complement, rather than replace, supervised operative experience.

Future development in thoracic surgery should focus less on the adoption of technology as an end in itself and more on demonstrating where new platforms, training methods, and digital tools provide measurable benefit. Comparative studies should include clinically meaningful endpoints, standardized assessment of nodal dissection and operative quality, economic evaluation, and longer follow-up, while also extending beyond the highly selected early-stage populations that dominate current randomized evidence. The success of minimally invasive and robotic thoracic surgery will ultimately depend not only on what the technology makes technically possible, but on how appropriately it is selected, taught, evaluated, and integrated into high-quality oncological care.

## Figures and Tables

**Table 1:** Main technical characteristics of the principal thoracic surgical approaches discussed in this review.

Surgical Approach	Main Advantages	Main Limitations	Current Role
Open thoracotomy	Wide exposure; tactile feedback; direct vascular control; facilitates complex reconstruction.	Greater access trauma; rib spreading; more postoperative pain and slower functional recovery.	Complex tumours and reconstructions; selected post-induction resections; situations in which exposure or vascular control outweighs access-related morbidity.
Multiportal VATS	Established minimally invasive approach; reduced access trauma; mature clinical and oncological experience.	Rigid instruments; commonly two-dimensional visualization, although 3D systems are available; limited direct tactile feedback; procedure-specific learning curve.	Standard minimally invasive route for many pulmonary resections in suitable anatomy.
Uniportal VATS	Single intercostal access; coaxial operative view; potential reduction in chest-wall trauma.	Instrument crowding; demanding stapler geometry; strong dependence on assistant exposure and camera handling; evidence remains predominantly observational.	Experienced VATS centres; selected lobectomies and segmentectomies; structured/proctored programmes.
RATS	Three-dimensional visualization; articulated instruments; surgeon-controlled camera; favourable ergonomics; useful for hilar and nodal dissection.	Higher platform-related costs; absent/limited haptic feedback; docking and team workflow; platform-specific training requirements.	Minimally invasive anatomical resections, including selected technically complex dissections, in established robotic programmes.

Abbreviations: 3D, three-dimensional; RATS, robot-assisted thoracic surgery; VATS, video-assisted thoracic surgery.

**Table 2:** Pivotal and high-level evidence supporting the clinical validation of minimally invasive lung resection.

Study	Design/Population	Comparison	Main Contribution	Main Limitation
Swanson et al., 2007 [4]	Prospective multicentre feasibility study; N = 128 enrolled; 111 stage I	VATS lobectomy feasibility	96/111 stage I patients underwent successful VATS lobectomy; demonstrated multicentre feasibility.	No open-surgery comparator; feasibility rather than comparative effectiveness.
Bendixen et al., 2016 [5]	RCT; N = 206; early-stage lung cancer	VATS vs. anterolateral thoracotomy	Less postoperative pain and better quality of life after VATS.	Patient-centred outcomes; not powered for long-term survival.
Long et al., 2018 [6]	Multicentre RCT; N = 508 recruited, N = 425 evaluable	VATS vs. axillary thoracotomy	Lower blood loss and shorter operative time with VATS; similar nodal yield, margins, morbidity and mortality.	Short-term endpoints.
VIOLET, 2022 [7]	Multicentre RCT; N = 503	VATS vs. open lobectomy	Better early physical recovery, shorter stay and fewer serious adverse events with VATS; comparable surgical quality.	Predominantly early-stage trial-eligible population; survival not primary endpoint.
ROMAN, 2021 [11]	Multicentre RCT; 77 randomized, N = 71 analysed	RATS vs. VATS	No broad perioperative superiority; more extensive nodal assessment in selected robotic metrics.	Small trial; stopped early.
RVlob short-term, 2022 [12]	RCT; N = 320	RATS vs. VATS lobectomy	Comparable short-term safety/recovery; selected technical differences; higher robotic hospitalization costs.	Single-centre trial; short-term analysis.
RAVAL, 2023 [13]	Multicentre RCT; N = 186 randomized, N = 164 analysed	RATS vs. VATS lobectomy	Comparable health utility; greater sampled LN count; ICER about USD 14,926/QALY in trial model.	Economic conclusions depend on healthcare context.
RVlob long-term, 2024 [14]	RCT; N = 320; median follow-up ~58 months	RATS vs. VATS lobectomy	RATS met non-inferiority criterion for OS; DFS comparable.	Not evidence of survival superiority.
Harris et al., 2026 [8]	IPD meta-analysis of 3 RCTs; N = 1185	VATS vs. open lobectomy	OS HR 0.79 favouring VATS; DFS HR 0.91, similar between approaches.	Primarily trial-eligible early-stage patients.
Ren et al., 2026 [15]	Systematic review/meta-analysis of 5 RCTs; N = 712	RATS vs. VATS	Lower blood loss and greater LN yield with RATS; no significant difference in conversion, overall complications, operative time or 3-year OS; higher costs.	Small randomized evidence base; heterogeneity among trials.

**Abbreviations:** DFS, disease-free survival; HR, hazard ratio; ICER, incremental cost-effectiveness ratio; IPD, individual-patient data; LN, lymph node; OS, overall survival; QALY, quality-adjusted life-year; RATS, robot-assisted thoracic surgery; RCT, randomized controlled trial; USD, United States dollars; VATS, video-assisted thoracic surgery.

**Table 3:** Main simulation modalities in thoracic surgical training and interpretation of the supporting evidence.

Modality/Example	Principal Training Target	Validation/Educational Evidence	Demonstrated Outcome	Evidence of Clinical Transfer	Main Limitation
Basic box trainer	Hand-eye coordination, instrument handling, suturing	Extensive MIS training experience; limited thoracic specificity.	Improvement in basic technical tasks.	Transfer evidence derives mainly from non-thoracic surgery.	Low anatomical fidelity.
Iwasaki/Fukuoka anatomical trainer [28]	VATS lobectomy steps	User evaluation and performance differences related to operative experience.	Procedure-specific practice and technical assessment.	Not directly demonstrated.	Early model; limited formal validation.
PVA hydrogel model [29]	VATS dissection, stapling, tissue handling	Anatomical and face realism described.	Procedure-specific practice.	Not demonstrated.	Cost/production and limited outcome validation.
Stupnik and related dry-lab models [30,40]	VATS lobectomy/segmentectomy, stapling, vascular dissection	Used within structured thoracic training programmes.	A programme using Stupnik plus Crabtree models improved objective dexterity metrics.	Operating room/patient transfer not established.	Small training cohorts; effect cannot be attributed to one simulator alone.
VATS VR simulator [32]	Lobectomy performance and competency assessment	Validity evidence; discrimination between experience levels.	Reliable simulator-based assessment.	Clinical transfer incompletely established.	Virtual tissue behaviour.
Robotic VR platforms [34,35,36]	Basic console skills	Randomized/meta-analytic evidence for skill acquisition.	Better objective scores and shorter task times versus no additional training.	Transfer evidence present but heterogeneous.	Most evidence is not thoracic-specific.
Sensorized vascular simulator [37]	Force control and vascular manipulation	Preliminary face/content/construct validation.	Objective force measurement and feedback.	Not demonstrated.	Preliminary validation; small cohort.
Wet-lab/animal models [31]	Tissue handling and complete procedural sequence	Long educational experience.	High procedural fidelity.	Direct comparative evidence limited.	Cost, availability, ethical/logistical requirements.
Multidisciplinary robotic simulation [44]	Teamwork, docking, emergency response	Emerging thoracic evidence.	Team/process training and system familiarization.	Patient-level effect not established.	Lack of standardized clinical endpoints.

**Abbreviations:** MIS, minimally invasive surgery; PVA, polyvinyl alcohol; VATS, video-assisted thoracic surgery; VR, virtual reality. Note: simulator validity, improvement in simulator performance, transfer to non-virtual tasks, operating room performance, and patient-level benefit represent different levels of evidence and should not be treated as interchangeable.

**Table 4:** Main short-term, technical, nodal, and economic outcomes after lung resection for NSCLC.

Outcome	Open vs. VATS	VATS vs. RATS	Interpretation
Pain/functional recovery	Randomized evidence favours VATS [5,7].	Differences are smaller and inconsistent across randomized RATS-VATS comparisons.	The strongest established access-related benefit concerns VATS versus thoracotomy.
Hospital stay	Shorter after VATS in VIOLET [7].	Generally comparable in randomized evidence.	Strongly influenced by ERAS and institutional pathways.
Operative time	Results vary across trials [6,47].	No significant pooled difference in RCT-only meta-analysis [15].	Poor standalone marker of operative quality.
Blood loss	Generally low with minimally invasive surgery.	Modestly lower with RATS in RVlob/pooling [12,15].	Statistical differences may be clinically small in routine cases.
Conversion	Not directly applicable as a VATS/open comparative endpoint.	No significant difference in pooled randomized evidence [15].	Influenced by selection, anatomy and experience; conversion may be an appropriate safety decision.
Overall complications	Contemporary randomized/comparative evidence favours minimally invasive access over thoracotomy [7,46].	Comparable RATS vs. VATS in pooled RCTs [15].	Access route is only one determinant of morbidity.
Lymph node yield/stations	Similar nodal upstaging after VATS and open surgery in VIOLET [7].	Some RCTs show greater yield/stations with RATS [11,12,13,15].	Node count alone does not define lymphadenectomy quality; anatomical extent/stations remain essential [1,2].
Nodal upstaging	VIOLET: similar VATS/open; broader comparative evidence remains heterogeneous.	Network meta-analysis suggests approach-related differences, but evidence is largely non-randomized [50].	Surrogate influenced by preoperative staging, pathology and case mix.
Downstaging after neoadjuvant therapy	Not an intrinsic access-related endpoint.	Comparable RATS/VATS in a selected retrospective post-chemoimmunotherapy cohort [48].	Primarily reflects treatment response and tumour biology.
Costs	VATS was cost-effective in the VIOLET UK NHS model, with numerically lower 12-month total costs and higher QALYs [47].	Direct/hospital costs generally higher with RATS; RAVAL found potential cost-effectiveness within its model [13,15].	Highly dependent on healthcare system, platform utilization and costing perspective.

**Abbreviations:** ERAS, Enhanced Recovery After Surgery; LN, lymph node; NHS, National Health Service; QALY, quality-adjusted life-year; RATS, robot-assisted thoracic surgery; RCT, randomized controlled trial; UK, United Kingdom; VATS, video-assisted thoracic surgery.

**Table 5:** Representative evidence on long-term oncological outcomes according to surgical approach in NSCLC.

Study	Design/N	Stage/Population	Comparison	Oncological Endpoint	Main Result	Main Limitation
Harris et al., 2026 [8]	IPD meta-analysis of 3 RCTs; N = 1185	Clinical early-stage NSCLC	VATS vs. open lobectomy	OS, DFS	OS favoured VATS (HR 0.79); DFS similar (HR 0.91).	Applies primarily to trial-eligible early-stage patients.
Niu et al., 2024 (RVlob) [14]	RCT; N = 320; median follow-up ~58 months	Resectable stage I-IIIA NSCLC	RATS vs. VATS	3-year OS, 3-year DFS	RATS non-inferior for OS; DFS comparable.	Single-centre trial; not evidence of superiority.
Yang et al., 2017 [53]	Retrospective propensity-matched cohort	Clinical stage I NSCLC	RATS vs. VATS vs. open	5-y OS, DFS	Similar OS; unadjusted DFS difference RATS vs. VATS, but approach not independently associated with survival.	Residual confounding; retrospective.
Ng et al., 2019 [51]	Systematic review/meta-analysis; 145 studies	Predominantly resectable NSCLC	VATS/open and MIS comparisons	OS, recurrence, nodal outcomes	Observational pooled evidence generally supported VATS/MIS oncological acceptability.	Predominantly non-randomized evidence; substantial heterogeneity.
Leitao et al., 2023 (RECOURSE) [55]	Systematic review/meta-analysis; lung lobectomy subgroup within multi-cancer analysis	Lung cancer subgroup	RATS vs. VATS/open	OS, DFS	Signals favouring RATS in selected comparisons.	Non-randomized evidence; heterogeneous multi-cancer framework.
Zirafa et al., 2023 [54]	Retrospective single-centre; N = 131	Locally advanced NSCLC	RATS vs. open	5-y OS, local RFS, metastasis-free survival	5-y OS ~34% vs. 31%; no significant recurrence/survival differences.	Selection bias; expert single-centre experience.

**Abbreviations:** DFS, disease-free survival; HR, hazard ratio; IPD, individual-patient data; MIS, minimally invasive surgery; NSCLC, non-small cell lung cancer; OS, overall survival; RATS, robot-assisted thoracic surgery; RCT, randomized controlled trial; RFS, recurrence-free survival; VATS, video-assisted thoracic surgery.

## Data Availability

No new data were created or analyzed in this study. Data sharing is not applicable to this article.
